# FIGO call to action: Multisectoral approach to postpartum hemorrhage

**DOI:** 10.1002/ijgo.70449

**Published:** 2025-08-21

**Authors:** Diana Ramasauskaite, Dietmar Schlembach, Akaninyene E. Ubom, Albaro J. Nieto‐Calvache, Cherrie Evans, Ahmet M. Gülmezoglu, Jolly Beyeza‐Kashesya, Bo Jacobsson, Alison Wright, For the FIGO Committee on Childbirth and PPH

**Affiliations:** ^1^ Clinic of Obstetrics and Gynecology Vilnius University Faculty of Medicine Vilnius Lithuania; ^2^ Clinic of Obstetric Medicine Vivantes Clinicum Neukoelln Berlin Germany; ^3^ Department of Obstetrics, Gynaecology and Perinatology Obafemi Awolowo University Teaching Hospitals Complex Ile‐Ife Nigeria; ^4^ Departamento de Ginecología y Obstetricia Fundación Valle del Lili Cali Colombia; ^5^ Facultad de Ciencias de la Salud Universidad ICESI Cali Colombia; ^6^ Department of Global Program and Technical Excellence, Jhpiego Helping Mothers Survive Baltimore Maryland USA; ^7^ Concept Foundation Geneva Switzerland; ^8^ Mulago Specialised Women and Neonatal Hospital Kampala Uganda; ^9^ Department of Obstetrics and Gynecology, Sahlgrenska Academy University of Gothenburg Gothenburg Sweden; ^10^ Department of Obstetrics and Gynecology, Western Health Care Region Sahlgrenska University Hospital Gothenburg Sweden; ^11^ Division of Health Data and Digitalisation, Department of Genetics and Bioinformatics Institute of Public Health Oslo Norway; ^12^ Consultant Obstetrics & Gynaecology Royal Free Hospital London UK; ^13^ University College London UK; ^14^ International Federation of Gynecology and Obstetrics (FIGO) London UK

**Keywords:** management, multisectoral approach, postpartum hemorrhage

## Abstract

Maternal mortality from postpartum hemorrhage is not only a medical issue but a social one as well, dependent mainly on persistent inequities in global health and socioeconomic development. Research, evidence‐based clinical practice, and advocacy are the cornerstones of strategy in decreasing maternal mortality and near‐miss cases of postpartum hemorrhage. Multisectoral coordination is crucial in optimizing the usage of resources by avoiding duplication of inputs and activities.

## 1 INTRODUCTION

Postpartum hemorrhage (PPH) remains the leading cause of maternal mortality globally.^1^ The maternal mortality rate due to PPH is very low in high‐income countries (HICs), while women in low‐ and middle‐income countries (LMICs) continue to experience a disproportionate impact.^2^

Progress in reducing maternal mortality appears to have stalled over the past 5–10 years, and future projections through 2030 are concerning.^2^ A global strategy is urgently needed to reverse this situation. Various initiatives have been launched and implemented by different organizations, including WHO, United Nations Population Fund (UNFPA), International Federation of Gynecology and Obstetrics (FIGO), regional bodies such as the Asia & Oceania Federation of Obstetrics & Gynecology (AOFOG), African Federation of Obstetricians and Gynecologists (AFOG), and the Latin American Federation of Obstetrics and Gynecology Societies (FLASOG), as well as numerous non‐governmental organizations (NGOs) and others.

However, the situation remains unchanged.^2^ Souza et al.^3^ stated that the preventable deaths of women “are not solely due to biomedical complications of pregnancy, childbirth, and postpartum, but are also dependent on persistent inequities in global health and socioeconomic development”.

Maternal mortality from PPH is not only a medical issue, but a social one as well. The Global Strategy for Women's, Children's and Adolescents' Health by WHO offers a multisector approach: “recognizing that health‐enhancing factors, including nutrition, education, water, clean air, sanitation, hygiene, and infrastructure, are essential” to achieving Sustainable Development Goals (SDGs).^4^

The Global Summit on PPH was organized by WHO in 2023. The participants were from different sectors, including representatives from ministries of health, healthcare professions, representatives of research institutions and academia, professional associations, national and international NGOs, civil society organizations, donor agencies, and innovators from the pharmaceutical and medical devices industries, and the private sector. The result of this summit was the Roadmap to Combat Postpartum Hemorrhage (2023 and 2030), and “a multisectoral approach was highlighted as being the most appropriate to solve this issue”.^2^ The participants of this summit identified that all stakeholders from different sectors—“health, environment, and economy”—should cooperate closely to reach the goal of reducing maternal mortality from PPH successfully.^2^ All stakeholders from various sectors—by contributing their knowledge, key areas of expertise, and resources, and “benefiting from their combined and varied strengths”—can achieve the goal more effectively.^5^

This manuscript serves as both a position statement and a best practice paper from the Committee on Childbirth and Postpartum Hemorrhage of FIGO, one of the leading organizations dedicated to driving effective and coordinated efforts aimed at eliminating preventable deaths caused by PPH. The article discusses critical gaps and identifies failures in preventing adverse outcomes associated with PPH.

It is a call for coordinated action to optimize the use of resources by minimizing duplication of inputs and activities.

## 2 DEFINITION AND INCIDENCE OF PPH

PPH is an obstetric emergency that complicates 1%–10% of all deliveries.^6^ These differences in reported rates are due to different case definitions of PPH and methods of measuring blood loss with visual estimation, often leading to significant underestimation. There are also significant gaps in awareness, training, and other factors leading to different reported numbers.

The most commonly accepted definition of PPH is blood loss from the genital tract of 500 mL or more after vaginal delivery, or 1000 mL or more after cesarean delivery, depending on the mode of birth.^7^

In 2014, Sheldon et al.^8^ published findings from the WHO Multicountry Survey on Maternal and Newborn Health. They analyzed the data from 352 health facilities in 28 countries and reported the rate of PPH as 1.2%. This reported incidence of PPH in this study is significantly lower than that found in other studies,^6^, ^7^, ^9^, ^10^ with reports in the range of 5.4%–14.22%. The authors concluded that this finding was most likely influenced by the use of visual assessment of postpartum bleeding, which was the predominant method for diagnosing PPH in the facilities surveyed. Other possible reasons include the reliance on measured blood loss alone when recording PPH incidence in studies, and the tendency of many providers in this survey to document only cases of severe PPH (clinically defined as blood loss ≥1000 mL).^8^

Borovac‐Pinheiro et al.^7^ suggested that a more accurate definition of PPH should include both blood loss and clinical signs of cardiovascular changes after delivery. In addition, some guidelines recommend using the shock index for a more accurate diagnosis of PPH.^7^, ^11^

No less important is the need to analyze not only the most serious events—maternal death—but also “near‐miss” cases. In the previously referenced Multicountry Survey on Maternal and Newborn Health,^8^ despite the high overall rate of prophylaxis (95.3% of all included women received uterotonic prophylaxis), 17.2% of PPH cases resulted in severe maternal outcomes— 14.5% were classified as near misses and 3.1% resulted in maternal death.

There are variations in PPH incidence and in the reported numbers of the most serious events—not only between regions and countries, but also among hospitals within the same country, even when all have the capacity to provide essential interventions.^1^, ^12^

The definition of PPH is one of the most critical gaps, as any discussion of a clinical condition should start with an evidence‐based definition to ensure a shared understanding. Other gaps, such as a lack of supplies and resources, lack of personnel training, and failures in the processes, are discussed elsewhere in this manuscript.

*CALL TO ACTION*: A uniform definition of PPH should be accepted worldwide and the rate of PPH should be monitored using the same criteria.

## 3 STRATEGIC ACTION PLAN TO REDUCE PPH

The situation varies across settings, highlighting the need for each country and institution to develop an action plan to identify weak points and implement measures for improvement, in line with the strategic plan proposed in the Roadmap.^2^

The Roadmap and Call to Action on PPH for the global maternal health community is organized around four strategic areas that work synergistically to create a profound and sustained impact in combatting PPH:^2^

- Research, which is necessary for establishing the evidence base for new interventions;
- Standards and norms, which include guidance recommending for or against a specific interventions, along with tools to support implementation;
- Implementation, which focuses on how recommended interventions are translated into clinical practice;
- Advocacy, the most critical component, which ensures that the process from research to practice is successfully carried out.

*CALL TO ACTION*: Given the varying situations regarding PPH in different settings, it is essential to develop an action plan for each country and institution.

## 4 PPH RESEARCH PRIORITIES

Aligning priority PPH research gaps along three tracks—innovation, implementation, and cross‐cutting—was identified as essential for focusing investments, reducing research duplication, and shortening the time needed to meaningfully address public health needs.^13^ In 2024 alone, 32 systematic reviews on PPH management were published, highlighting the need for better coordination of research and more rational use of the collective potential of scientists.

Williams et al.^13^ published a manuscript describing a WHO‐led effort to develop a global PPH research agenda for 2023–2030, to avoid duplication and waste. The three research domains related to the burden of PPH are assessment, development of new capacities, and improvement of existing capacities for reducing PPH. Five scoring criteria for setting research priorities—answerability, effectiveness, deliverability, potential impact, and equity—were also highlighted.^13^

The new strategy was implemented with the involvement of representatives from women's organizations in the research prioritization process. In the innovation section, the highest priority was assigned to research on the non‐intravenous administration of tranexamic acid for treating PPH. In the implementation section, the main research focus is on identifying gaps in the adoption and use of guidelines for managing PPH. In the cross‐cutting section, priority was given to research evaluating the effectiveness of early diagnosis and first‐line treatment of PPH in improving outcomes.^13^ Furthermore, WHO will develop detailed Target Policy Profiles^14^ to support funding for proposals addressing the most prioritized questions, rather than studies considered irrational or irrelevant.

*CALL TO ACTION*: It is necessary to coordinate research and utilize the collective potential of scientists more effectively, as well as to provide funding for proposals on prioritized topics.

## 5 GUIDELINES AND RECOMMENDATIONS FOR THE PREVENTION AND TREATMENT OF PPH

WHO and other global organizations have updated and published several evidence‐informed recommendations for the prevention and treatment of PPH.

Giouleka et al.^15^ published a descriptive review of guidelines on PPH evaluation from the following bodies: the American College of Obstetricians and Gynecologists; the Royal College of Obstetricians and Gynaecologists; the Royal Australian and New Zealand College of Obstetricians and Gynaecologists; the Society of Obstetricians and Gynaecologists of Canada; the Network for the Advancement of Patient Blood Management, Hemostasis and Thrombosis (in collaboration with FIGO); the European Board and College of Obstetrics and Gynaecology; the European Society of Anaesthesiology; and WHO.

Establishing evidence‐based guidelines, particularly those adapted to LMICs, will be beneficial. Despite intense efforts to implement these recommendations, PPH remains the leading cause of maternal complications and death worldwide.

Gallos et al.^16^ identified several key challenges contributing to this failure: PPH is often undetected or detected too late, interventions are used inconsistently or delayed, and overall implementation is poor. In a large, randomized trial of prophylaxis for PPH, only 53% of the participants who developed PPH were diagnosed and treated with a uterotonic drug.^17^

In many institutions worldwide, blood loss at birth is assessed visually—a method widely recognized as inaccurate and prone to underestimating blood loss.^17^ PPH is a time‐critical condition, and delays in the use of lifesaving interventions can lead to death or severe morbidity.^16^, ^19^, ^20^

The first steps have been taken to address the challenges mentioned earlier.^18^ A modified Delphi‐based international expert consensus on strategies for optimizing early detection and obstetric first response management of PPH at cesarean birth was published in 2024.^21^ The key points from the expert consensus should be integrated into all recommendations:

- The definition of PPH is the same regardless of the mode of delivery;
- “Quantitative blood loss measurement, complemented by monitoring the woman's hemodynamic status,” is the first intervention in early detection of PPH;
- The first response when PPH is diagnosed (defined as blood loss of at least 500 mL with continued bleeding or clinical signs of hemodynamic instability, whichever occurs first) is the “immediate administration of uterotonics and tranexamic acid for the first response following the cause‐specific responses”.^21^

Most guidelines for managing and preventing PPH focus on interventions during labor, delivery, or the postpartum period. However, the prevalence of anemia during pregnancy—estimated at approximately 40%—is a significant risk factor for PPH.^22^ In addition, anemia due to menstrual disorders or other causes before pregnancy, as well as interventions to treat anemia before conception, should also be considered. The incidence of anemia varies across regions and is linked to socioeconomic status. Correcting anemia through a multisectoral approach—including education, screening, access to healthy food, and iron supplementation—is essential for improving outcomes. Such efforts can help reduce maternal mortality from PPH, particularly in LMICs.

*CALL TO ACTION*: Recommendations and clinical guidelines on the management of PPH should be based on high‐quality, evidence‐based research or the consensus of international experts and updated regularly. Recommendations for managing anemia preconceptionally should be included due to its significance as a risk factor for PPH.

## 6 IMPLEMENTATION OF RESEARCH AND RECOMMENDATIONS INTO CLINICAL PRACTICE

Key categories of implementation barriers were identified during the Global Summit organized by WHO in 2023: lack of clear national health policies and leadership; weak procurement and supply chain systems; widespread use of non–quality‐assured medicines; inadequate staffing, training, and supervision of health workers; inequities and limited access to good quality care; and women's limited rights and disadvantaged social status.^2^

### 6.1 Challenges in implementation arising from healthcare providers

Although recommendations for PPH management are widely available, a significant barrier to reducing severe complications lies in the effective implementation of these guidelines.

Several obstacles have been identified, including limited staffing, lack of relevant knowledge and skills, lack of engagement from healthcare providers, and professional attitudes that discourage task sharing.^16^, ^23^

Risk factors for PPH are identified in only approximately one‐third of cases before or during labor. These include a history of prior PPH, an overdistended uterus, placental abnormalities, coagulation disorders, severe anemia, factors related to labor itself (such as induction or augmentation), epidural anesthesia, prolonged labor, cesarean delivery, and other causes.^11^ Therefore, effective and timely prevention, diagnosis, and management of PPH should be available at every delivery. In Europe and other parts of the world, doctors and midwives work under time constraints; junior specialists in particular may not encounter rare obstetric emergencies frequently enough to gain or maintain competence.^24^

In addition, changing practices within obstetric and midwifery services, excessive workloads in LMICs, and a shortage of other specialists in rural areas raise concerns about how the multidisciplinary teams needed for PPH management can gain and maintain experience.^24^

Effective teamwork and communication among team members are crucial for improving PPH management and reducing adverse outcomes.^24^, ^25^ Evidence shows that traditional methods, such as case reporting and analysis, are often reactive and not always effective. Simulation‐based training offers a proactive approach to maintaining and improving both technical and non‐technical skills, reducing errors in emergency obstetrics, and enhancing teamwork and communication.^24^

Training should involve all members of the labor ward team and beyond, including hematology and non‐clinical staff, where available.^26^ During simulation training, respect for laboring women should be emphasized. Realism, relevance, repetition, and reflection are the most critical factors for the best training results. Evidence shows that it is beneficial to attend emergency simulation training every year.^25^ Simulation training in the local unit allows members of the team to practice in their workplace, to detect potential weaknesses in the organization and teamwork. Training costs can be reduced. Still, effectiveness can be influenced by the quality of teaching.^27^

Nelissen et al.^28^ showed that simulation‐based training was associated with a 38% reduction in the incidence of PPH.

Another key objective is to promote the rational use of health interventions and to prevent interventions that are not medically justified. Complications of health interventions and iatrogenic factors are an essential contributor to all‐cause mortality and a substantial cause of or contributory factor to maternal mortality.^29^, ^30^

A medically indicated cesarean section (CS) can be a life‐saving intervention. The underuse of CS can be associated with poor maternal and perinatal health outcomes.^31^ Health education, mandatory written informed consent, evidence‐based intrapartum care to promote a positive childbirth experience, the presence of support persons during birth, training for midwives and obstetricians to maintain skills for managing complicated deliveries, and improvements in emergency obstetric care procedures are all strategies described to reduce rates of unnecessary CS.^32^

Strengthening support for first responders within the health system is also a priority. Innovative strategies such as telemedicine have proven useful both in basic settings^33^ and complex situations in referral hospitals.^34^

### 6.2 Challenges in implementation arising from healthcare systems and local support from stakeholders

Maternal healthcare systems and health sector stakeholders play crucial roles in organizing and shaping the interrelated forces and contexts discussed earlier. Health services and commodities can help modify the impact of socioeconomic factors that contribute to adverse maternal health outcomes.^3^

Therefore, a strong and resilient health system can be considered a decisive protective factor, capable of neutralizing or minimizing the effects of harmful risk factors. For example, the adverse effects of certain risk factors, such as advanced maternal age or low‐income status, can be mitigated by well‐functioning health services, particularly those offering high‐quality preconception, antenatal, intrapartum, and postpartum care.^3^

The most significant progress in strengthening health systems is attributed to effective country leadership that enables “meaningful collaboration between different arms of the government, working closely with the public and private sectors to achieve health targets”.^35^, ^36^ This is reflected in strong policy and law‐making, comprehensive legislation, a well‐equipped workforce, functioning infrastructure, adequate funding, robust data for decision‐making, and transparency and accountability.^35^, ^37^, ^38^ In contrast, countries with the highest mortality rates from PPH often have health systems with limited resources and infrastructure, a lack of political will, inadequate long‐term planning, poor emergency preparedness, and insufficient sustainable financing.^35^, ^38^, ^39^

A shift in the culture of obstetric care is urgently needed to better meet the needs of women giving birth. The focus should be on women, and their perspectives should be central to provision of care.

Policies and guidelines for safe obstetric care must be developed and effectively implemented. Healthcare systems and policymakers must work together with healthcare professionals to ensure an appropriate infrastructure (birth units, hospitals) within reasonable distance for all women. This includes appropriate emergency transport systems available in a timely manner, and, especially in regions with large distances, appropriate infrastructure allowing women at risk to stay near a (specialized) hospital or facility until delivery, ideally accompanied by her existing children.

The majority of maternal deaths due to PPH complications occur in LMIC settings. This disparity is largely attributed to differences in the quality of care, including the availability of trained personnel at deliveries, access to quality uterotonic drugs, and the timely administration of necessary interventions during obstetric emergencies.^7^

Oxytocin and tranexamic acid are listed on the WHO Essential Medicines List for the management of PPH.^39^ The reasons for the poor quality of the medications include substandard manufacturing, inadequate distribution and storage conditions, counterfeiting, or a combination of these factors.^40^

The acceptance criterion for oxytocin indicates “that the product contains no less than 90.0% and no more than 110.0% of the amount of active pharmaceutical ingredient” declared on the label. For tranexamic acid, the criterion is no less than 95.0% and no more than 105.0% of the active ingredient, without contaminants.^40^

A systematic review by Torloni et al.^41^ showed that 48.9% of 1890 uterotonic samples (from 19 studies) were substandard in LMICs, with 75% of ergometrine samples and nearly 40% of oxytocin and misoprostol samples failing quality standards. Ammerdorffer et al.^40^ published the first analysis of tranexamic acid quality in LMICs and found that all products met the acceptance criterion for active ingredient content; however, the presence of contaminants remains a concern. A significant advantage of tranexamic acid is that it does not require a cold chain to maintain product quality of the product; facilities typically store it between 19°C and 27°C.^40^

Heat‐stable carbetocin may serve as an alternative to oxytocin for the prevention of PPH;^42^ however, quality‐assured oxytocin for treatment is still required. Another critical factor is the appropriate storage and transportation of uterotonics (especially oxytocin) and blood products. Oxytocin should be stored in a cold chain at 2–8°C,^43^ although some manufacturers register oxytocin for storage at 15–25°C.^43^ Nguyen et al.^43^ found that these products have degradation profiles similar to those labeled for cold storage.^43^ Poor cold chain management poses “a potential risk to the loss of potency and ineffectiveness of the treatment”.^44^

An efficient cold chain system depends on three key elements: trained cold chain technicians, reliable storage, and proper transportation facilities.^44^ The main issues associated with inadequate cold chain management include old equipment, power outages, and uncalibrated refrigerators and temperature monitoring devices.^44^

Qualitative evidence conducted by Torloni et al.^45^ in 2023 suggests that many healthcare providers do not formally report suspected low‐quality oxytocin or misoprostol. Instead, they use higher doses or additional uterotonics to compensate. The medication's expiration date should be checked before every administration, and expired products should not be used.

A systematic review conducted by Ginnane et al.^46^ in 2024 examined the cost‐effectiveness evidence for postpartum interventions to prevent, diagnose, or treat PPH. The authors concluded that both tranexamic acid as part of PPH treatment and “comprehensive PPH bundles for prevention, diagnosis, and treatment have supportive cost‐effectiveness evidence across a range of settings”.^46^ Additional priority interventions recommended by WHO, such as uterotonics, the non‐pneumatic anti‐shock garment, and uterine balloon tamponade, require robus economic evaluations across high‐, middle‐, and low‐resource settings.^46^

The healthcare system should be prepared for disease outbreaks, conflicts, and other public health emergencies that aggravate the situation by increasing the risk of pregnancy complications, disrupting health systems, and posing additional constraints to maternal healthcare.^47^

*CALL TO ACTION*: Clear national health policy and leadership, collaboration between different arms of the government, sufficient funding, transparency and accountability, training and supervision of health workers are essential in the implementation of PPH recommendations and improving the outcomes.

## 7 ADVOCACY, CALL TO ACTION, AND PUBLIC AWARENESS

Strong advocacy is needed across all levels to elevate PPH on global political agendas.^2^ Advocacy should raise awareness about the importance of timely and effective management of PPH, ensure that the health workforce is trained to be competent and confident in the prevention and management of PPH, that healthcare facilities providing birthing care have the necessary supplies and equipment, and that policies and guidelines prioritize maternal health and safety.^2^

All stakeholders should be engaged and work synergistically, and women should be at the center of the advocacy agenda. Women's groups and movements should be involved in advocacy, which can help drive attention to PPH and hold governments accountable.^2^ Advocacy activities can include a global “PPH Day,” regional conferences, and other events. Sustainable financing for PPH advocacy is critical to achieving the goals.

Patient education about the risk factors, prevention, and treatment of PPH is essential to achieve awareness, especially for women who are at high risk.^3^

During the advocacy process, attention should be given not only to medical issues but also to the social determinants of health—including economic, political, and cultural superdeterminants. These determinants encompass the conditions in which women are born, grow, work, and live before pregnancy, during pregnancy, labor, and postpartum. They are indirectly responsible for the disparities in maternal mortality and morbidity rates observed across different populations.^3^

A cross‐sectional study across 29 countries in Africa, Asia, Latin America, and the Middle East found that women with lower levels of education face a higher risk of severe maternal outcomes, even after adjusting for key confounding factors.^48^ Other important social determinants include low income and socioeconomic status, ethnic and racial dynamics that perpetuate racism and discrimination, exposure to sources of misinformation, living in rural areas, hunger, corruption, armed conflict, and violence.^36^

The use of more innovative approaches for advocacy, such as social media, publication programs targeted to specific user groups, seminars, eLearning platforms, live chat sessions, and podcasts, was favored by higher‐income countries.

Patient organizations, such as the European Foundation for the Care of Newborn Infants (EFCNI), can play a huge role in advocacy.

Some LMICs favored more traditional approaches, such as press conferences, traditional media appearances, general awareness campaigns, presentations, seminars, and launch events.^49^

*CALL TO ACTION*: All stakeholders should be engaged and use all approaches for PPH advocacy. Sustainable financing for PPH advocacy is critical to achieving the goals.

## 8 MULTISECTORAL APPROACH

Policymakers and stakeholders, especially in countries with high maternal mortality, must recognize that the leading biomedical causes of preventable maternal deaths, including PPH, do not occur in isolation.

Multisectoral action to promote social development and gender equity is essential for achieving a sustainable reduction in maternal mortality.

Although the improvements in social infrastructure and other social transformation programs often take time, their longer‐term benefits are reasonably certain.^3^ Intersections between factors, particularly gender, ethnicity, and socioeconomic class, are highly relevant in determining inequities in income (which affects the quality of care a woman can access), education (which influences how informed she is about her choices during pregnancy and childbirth), and personal agency (which determines her ability to advocate for her needs).^49^

Multisectoral coordination is critical in achieving the SDGs^4^, ^50^ and includes:

1. Adopting a multisector approach to improving the health and well‐being of women, children, and adolescents. It is essential to identify and assess policies and interventions in various sectors, to identify potential health risks and potential solutions.
2. Building governance and capacity to facilitate multisector action and cross‐sector collaboration. Strengthening coordination, financing, and accountability mechanisms to manage multisector action, and eliminating bureaucratic and financial barriers to cross‐sector collaboration is vital, not only within governments, but also among international agencies, the private sector, and NGOs.
3. Monitoring the impact of multisector action and cross‐sector collaboration on health and sustainable development.

The recommendations for a multisectoral approach by the stakeholder group are displayed in Table 1.

**TABLE 1:** The recommendations for a multisectoral approach by stakeholder group.

Stakeholder group	Call to action
Professional organizations (FIGO, regional and country organizations of obstetricians, gynecologists, midwives, and others), healthcare organizations (WHO)	Coordination of a multisectoral approach Research prioritization Production of guidelines and recommendations based on evidence and high‐quality research, and their updating Recommendations for the implementation of guidelines into clinical practice and audit of implementation Advocacy
Healthcare providers	Implementation of the guidelines into clinical practice Continuous updating of knowledge and skills Submitting the proposals for research Making proposals for policymakers to improve the health system
Policymakers, representatives from the ministries of health	Coordination of collaboration between different arms of the government, including health, economy, social services, and others Development of policies and legislation Assurance of functioning infrastructure, sufficient sustainable financing for health systems Seeking funding to enhance social infrastructure and support various programs aimed at social transformation
Research institutions and academia	Initiation of research in a prioritized subject Educating and training healthcare system providers Advocacy
Non‐governmental organizations	Coordination in advocacy Taking part in research prioritization Fundraising for research, medical education
Pharmaceutical and medical device industries	Ensuring safe medicines and devices on the market Providing technical support for medical devices and equipment Training on the effective and safe use of medical devices and equipment for medical staff
Private sector	Raising funds for medical education, research, and social programs Advocacy

## 9 CONCLUSIONS

PPH remains the leading cause of maternal mortality. Deaths from PPH are largely preventable and are not solely dependent on purely medical complications of pregnancy, childbirth, and postpartum, but are dependent on persistent inequities in global health and socioeconomic development.

Gender disparities, income, education, ethnicity, and race are strong predictors of death and disability during pregnancy, childbirth, and postpartum.

Research, evidence‐based clinical practice, and advocacy are the cornerstones of strategy in decreasing maternal mortality and near‐miss cases of PPH.

Multisectoral coordination and collaboration are crucial to optimize use of resources, reduce PPH, and ultimately improve care for women globally, both during childbirth and beyond.

## AUTHOR CONTRIBUTIONS

DR and DS contributed to the study conceptualization, planning and design. DR, DS, AEU, AJN‐C, CE, and AMG contributed to the literature search, review, and synthesis. DR wrote the first manuscript draft. DS, AEU, AJN‐C, CE, AMG, J‐BK, BJ and AW reviewed, revised, and edited the first manuscript draft for sound intellectual, contemporary, and best‐evidence content. AW coordinated the reviews, revisions, and edits while DR implemented them to produce a final manuscript. All authors read and agreed with the final manuscript.

## FUNDING INFORMATION

No funding was received.

## CONFLICT OF INTEREST STATEMENT

The authors have no conflicts of interest.

## Data Availability

Data sharing is not applicable to this article as no new data were created or analyzed in this study.
